# Supplementary material for: Mycetoma epidemiology, diagnosis management, and outcome in three hospital centres in Senegal from 2008 to 2018
Source: PLoS One. 2020 Apr 24;15(4):e0231871. doi: 10.1371/journal.pone.0231871 (PMC7182189; doi:10.1371/journal.pone.0231871)
Supplement: Attachment — Submitted filename: Responses to reviewers comments PNTD-sr.docx [file pone.0231871.s001.docx]

**To the Editorial Board of Plos Neglected Tropical Disease**

**Revision of submission PNTD-D-19-01698**

**Responses to editor and reviewers.**

**Editor comment**:

I am wondering if the authors tested any of the samples for a particular mycetoma agent circulated in Senegal via molecular diagnostic techniques; I think it is important to consider this point by approaching any of our colleagues at Erasmus Medical Research Institute.

1. **Response**: **The study performed here was a retrospective study, looking back at the records of the hospital centers in Senegal. During that time molecular identification of the causative agents was not performed and the grains were not stored. However, we plan to start prospectively collecting and storing the samples from diagnosed cases for molecular diagnosis purposes. So, we will contact the colleagues at Erasmus or other reference center for possible collaboration in this project.**

I would also ask the authors to look carefully for the previous literature in the region, particularly the ones related to distribution and prevalence of mycetoma (e.g. Van de Sande 2013, and Samy et al. 2014).

2. **Response**: **The information described in one of these paper (Van de Sande 2013) have been used to describe the background of the study and to discuss some results (Reference # 2). Samy *et al*. (2014) refers to ecological data that has not been collected for this study.**

**METHODS**

Reviewer #2: The study population is clear and the methodology used sound. For species identification I would like to see molecular verification though.

**Response**: **See response number 1**

**RESULTS:**

Reviewer #2: The results and figures are clear. However since the majority of the eumycetoma cases are treated with terbinafin and not with itraconazole which is much more used in other endemic countries the manuscript would benefit with an extra sub-analysis of this. Could the authors provide a more in depth analysis of the therapeutic outcome of the 68 patients treated with terbinafine and surgery. How was the treatment scedule. What dosages were used? Did you have higher success rates in smaller lesions? What about the otucome per species. Did you see differences between madurella cases and falciformispora cases.

3. **Response**: “**The patients with eumycetoma cases were treated with terbinafine 500 mg twice daily for 24 – 48 weeks. Among the 68 patients treated with terbinafine, information on the outcome was available only for 22 patients. The other patients were lost to follow-up. Among these 22, 20 patients recovered completely and two patients relapsed. In the 20 patients treated with terbinafine who recovered, one patient had a small lesion (less than 5 cm), 4 patients had lesions between 5-10 cm, and 10 patients had lesions larger than 10 cm.**

**Terbinafine treatment was combined with surgery in 49 of the 68 patients. Among these patients, 31 were treated with surgical removal and 18 were amputated. The 20 patients who recovered had been treated with terbinafine combined with surgery. Ten of them were treated with surgical removal and 10 were amputated. No patient has been cured with terbinafine only.”**

**Theses clarifications have been added in the manuscript.**

**Little information was available on the outcome per species. In the 20 patients successful cured with terbinafine, only three cases of *M mycetomatis* have been correctly identified. The other patients were treated based on the color of the grain.**

Reviewer #2: Furthermore you mention that traditional phytotherapy is used in the majority of the patients. Can you give a bit more details on the plants they used and how long. Was the traditional phytotherapy stopped or continued when antibacterial or antifungal therapy was started?

4. **Response**: **Information on the phytotherapy was collected at the interview. Patients have just reported a history of plants use before their visit to the clinic. They were not able to give details on the name of the plants, the treatment schedule or the precise duration of the treatment. In general, they stop the traditional phytotherapy when they start the antimicrobial therapy. However, more studies are needed to respond to these different questions.**

**CONCLUSIONS:**

Reviewer #2: The limitations on species identification should be mentioned. Also why only half of the patients was confirmed need some attention in the discussion

5. **Response**: **The limitation regarding fungal species identification has been added in the conclusion. This aspect on the difficulty to confirm the cases has been added in the discussion section.**

**EDITORIAL AND DATA PRESENTATION MODIFICATIONS**

Reviewer #2: The grammar used in the manuscript is not always correct. Change the following: Line 83-84: with the formation of a tumor-like

Line 85: replace various microorganisms by the causative agent

Line 98: It was added to the list of NTDs in may 2016 not in February 2013.

Line 189: associated with amoxicillini??? What do you mean with associated with? Supplemented?

Table 3: replace the word antibacterial with medical treatment or antibiotics. You mention both antibacterial and antifungal agents under this same line. Lower terbinafine to the lower part of the table. So that you first have the antibacterials, than the antifungals. Was itraconazole used alone as well?

6. **Response**: **All these corrections have been made as recommended. Itraconazole was not used alone.**

Reviewer #2: Furthermore the text in lines 176-182 was not clear. I did not understand the meaning. Could you include what you mean with diagnosis of mycetoma was confirmed in 97 cases. Do you mean confirmation by culture and/or histology. In the next sentence you mention that only in 29% (59 cases) of all cases you obtained an isolate. Since you speak of both bacteria and fungi I assume mycological examinations is in fact microbiological examinations. Furthermore in line 181-182 you mention confirmation in 95 cases. What is the true figure?

7. **Response**: **This part has been clarified in the text. The true figure is 97 cases confirmed by culture and/or histology**

**SUMMARY AND GENERAL COMMENTS**

Reviewer #1: It is an important series that gives us precise data about another African region of mycetoma. In general the data is interesting and well analyzed.

The most important thing is that they give an explanation in the cases that were undetermined, how were they diagnosed? if it is for the grains they can at least be classified into actino or eumycetomas

Some of my suggestions are as follows.

Name change of fungal names: Falciformispora senegalensis and Pseudallescheria boydii (anamorphic: Scedosporium boydii/Scedosporium boydii)

Don't use Bactrim because it's a brand

8. **Response**: **The diagnosis of undetermined cases was mostly based on grain color (yellow and white). The microbiological examinations and histology yielded negative results. As both actinomycetoma and eumycetoma agents can give yellow and white grains, it was difficult to classify them in one of the two groups. This precision has been added in the manuscript**

**The names of the agents have been changed as recommended.**

Reviewer #2: The manuscripts describes the current epidemiology of mycetoma in senegal. This is data currently lacking in literature and therefore essential. Additionally the patients treated in senegal are treated with other antifungal agents than countries more regularly reporting on mycetoma. It would therefore be nice do include a sub-analysis in which the treatment oucome is analysed in more detail. In this patient an analysis of 68 patients can be made. That is the biggest cohort of eumycetoma patients treated with terbinafine and would be very valuable data.

9. **Response**: **See response 3**
